# Triggered Rapid Degradation of Nanoparticles for Gene Delivery

**DOI:** 10.1155/2012/291219

**Published:** 2012-06-19

**Authors:** José M. Morachis, Enas A. Mahmoud, Jagadis Sankaranarayanan, Adah Almutairi

**Affiliations:** ^1^Skaggs School of Pharmacy and Pharmaceutical Sciences, University of California at San Diego, 9500 Gilman Drive, MC 0600 La Jolla, CA 92093-0657, USA; ^2^Materials Science and Engineering Program, University of California at San Diego, 9500 Gilman Drive, MC 0600 La Jolla, CA 92093-0657, USA

## Abstract

Effective gene delivery tools offer the possibility of addressing multiple diseases; current strategies rely on viruses or polyplexes. Encapsulation of DNA within nanoparticles is an attractive alternative method for gene delivery. We investigated the use of our recently developed Logic Gate Nanoparticle for gene delivery. The nanoparticles, composed of a dual pH response random copolymer (poly-**β**-aminoester ketal-2), can undergo a two-step “in series” response to endosomal pH. The first sep is a hydrophobic-hydrophilic switch, which is followed immediately by rapid degradation. Rapid fragmentation is known to increase cytoplasmic delivery from nanoparticles. Therefore, we hypothesized that our Logic Gate Nanoparticles would enable increased gene delivery and expression relative to nanoparticles that degrade more slowly such as PLGA-based nanoparticles. Passive nanoparticle entry into cells was demonstrated by delivering Cy5-labeled pDNA encoding EGFP into HCT116, a colon carcinoma cell line. Flow cytometry analysis showed that cells are positive for Cy5-DNA-nanoparticles and produced EGFP expression superior to PLGA nanoparticles. Inhibition of V-ATPases using bafilomycin A1 demonstrates that expression of EGFP is dependent on low endosomal pH. The advanced Logic Gate Nanoparticles offer new therapeutic possibilities in gene delivery and other applications where rapid release is important.

## 1. Introduction

Emerging gene delivery tools offer novel therapeutic approaches to address several types of diseases including progeria, cystic fibrosis, Parkinson's, and multiple types of cancers. Gene therapy encompasses the entire process of effectively delivering functional DNA into cells to replace a missing or mutated gene within malfunctioning cells. One of the main challenges with gene delivery is that free DNA circulating in the body is exposed to nuclease degradation. Additional obstacles for gene delivery include the inability of DNA to cross the cell membrane, escape the endosome, and enter the nucleus due to the DNA's size and negative charge. Though virus-mediated delivery of DNA offers high transfection efficiencies and high expression rates [1], viral vectors face several fundamental problems including toxicity, immunogenicity, and high manufacturing costs [2, 3].

Nonviral polymeric systems offer an attractive alternative to deliver plasmid DNA and other nucleic acid molecules like siRNA, as they are generally less immunogenic [4–7]. However, polymeric systems must overcome various challenges to induce gene expression. In order to promote high efficiency of gene delivery, DNA must escape from the endosome before degrading within the late endosome and lysosome. A method that is widely used to promote endosomal lysis is to include chloroquine within the formulation [8]. A drawback of chloroquine, however, is that it can disrupt potentially all the endosomes and lysosomes in the cell [9, 10]. Advances in cationic polymers such as poly (L-lysine) (PLL) and polyethyleneimine (PEI), PAMAM dendrimers, and chitosan have shown some promise in complexing DNA into polyplexes and use for DNA delivery in vivo [11–15]. The positively charged complexes allow binding and entry into the negatively charged cell membrane. Additionally, the large number of amino groups in PEI and other polymers offers a buffering effect in the low pH environment of the endosome and creates a “proton sponge” leading to endosomal burst and release of the DNA complex into the cytoplasm to produce higher transfection efficiencies [16].

Because polyplexes cause toxicity and are relatively unstable, nano- and micro-particles provide an alternative method for delivery. Nanoparticles provide superior protection from circulating nuclease activity and offer an array of possible targeting advantages when combined with specific peptides. Nanoparticles composed of synthetic polymers such as poly(lactic-co-glycolic acid) (PLGA) are safe and attractive methods for DNA delivery applications and have been used in several studies [17]. Encapsulation of DNA with PLGA protects it from nuclease degradation, but the DNA is released slowly over time as PLGA degrades through ester hydrolysis [18, 19]. An additional limitation of using PLGA nanoparticles is their negative charge that must be modified to reduce this barrier to DNA encapsulation and delivery [20].

**Figure 1:**
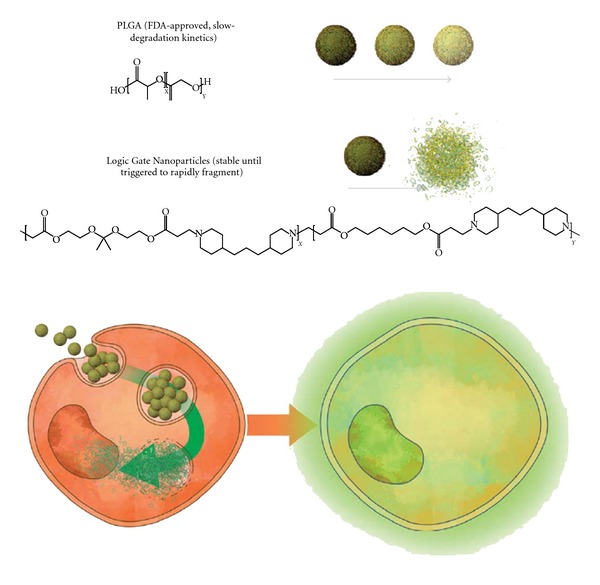
Schematic representation of the dual pH-responsive nanoparticles used for gene transfection.

In this paper, we investigated a novel gene delivery system using Logic Gate Nanoparticles developed with a dual pH-responsive random copolymer (poly-*β*-aminoester ketal-2, Figure 1) [21]. Current pH-responsive polymers have been demonstrated and are promising gene delivery systems [22]. However, our random copolymer is unique because it remains hydrophobic at physiological pH (pH 7.4) but undergoes a switch from hydrophobic to hydrophilic at low endosomal pH, which initiates rapid fragmentation into small molecules. The amine moieties in the backbone undergo a sharp hydrophobic-hydrophilic switch. This leads to an increase in water uptake (bulk dissolution) and hence an increase in ketal hydrolysis (degradation) [23]. The nanoparticle formulations are stable for 24 hours in physiological pH [21], as TEM revealed well-formed particles, and upon reducing the pH to endosomal levels, pH 5, these dual responsive nanoparticles undergo a rapid and dramatic fragmentation followed by concomitant release of their payloads (Figure 1). We hypothesized that these nanoparticles would be suitable for gene delivery and efficient gene expression. In this study, we demonstrate that nanoparticles composed of the dual pH-responsive polymer offer effective endosomal release and expression of encapsulated DNA due to its ability to undergo rapid fragmentation.

## 2. Materials and Methods

### 2.1. Materials

Dichloromethane (DCM, methylene chloride) and trehalose were purchased from Fisher Scientific (Hampton, NH, USA). Poly (vinyl alcohol) (PVA) (MW 30–70 k) and bafilomycin A1 were obtained from Sigma Chemical Co. (St. Louis, MO, USA). PLGA (Resomer RG 502H) was purchased from Boehringer Ingelheim (Germany). Cy5 labeling kit was obtained from Mirus Bio (Madison, WI, USA). Cell culture media was purchased from Life Technologies (Carlsbad, CA, USA). All reagents were purchased from commercial sources and were used without further purification unless otherwise stated.

### 2.2. Plasmid Preparation and Cy5 Labeling

 The pEGFP plasmid was expanded in overnight cultures of DH5 alpha *E. coli* cells and purified using maxi prep kits (Life Technologies). The DNA was Cy5-labeled using the Label IT Tracker Intracellular Nucleic Acid Localization Kit (Mirus, USA). In brief, the DNA plasmid was incubated with Label IT tracker reagent in the labeling reaction at 37°C for 1 hr. Then, labeled DNA was separated from free dye using Micro Bio-Spin 30 chromatography columns (BioRad, Hercules, CA, USA).

### 2.3. Synthesis and Characterization of Poly-*β*-Aminoester ketal-2

 Following the literature procedures and in agreement with previously described polymer characterization [21], the polymer was prepared by Michael addition of the corresponding diacrylates with trimethyl dipiperidine. Molecular weight was estimated by size exclusion chromatography against polystyrene standards in DMF/0.01% LiBr with a VWD (variable wavelength detector) at 250 nm. Mw = 6300, Mn = 2880, and PDI = 2.18.

### 2.4. Preparation of Nanoparticles

The nanoparticles were prepared using PLGA or the pH-responsive polymer using W/O/W method. In a vial, 10 mg of the polymer was dissolved in 300 *μ*L of DCM. Subsequently, 30 *μ*L DNA solution prepared in Tris-HCl buffer pH 8 was added. The two phases were sonicated for 30 s at 6 W (amplitude of 2, Misonix S-4000, 5.5′′ cup horn, USA). Then, an aqueous solution of 3 mL 1% PVA in Tris-HCl buffer pH 8 was added and sonicated for two 30 s cycles at 7 W (amplitude of 5) using the same cup horn. The nanoparticle suspension was stirred at 500 rpm under vacuum using a magnetic stirrer to evaporate DCM. A concentrated mode tangential flow filtration system using 500 kDa MicroKros modules (Spectrum Labs) was used to remove the PVA and free DNA [24]. The nanoparticle suspension was concentrated and washed two times. Finally, the suspension was lyophilized after adding 5% trehalose. The nanoparticle characterization and properties were in agreement with the previously described literature [21]. In brief, dynamic light scattering (DLS, Malvern Zetasizer) revealed that pH-responsive particles had Z-average diameters of 300 nm (PDI = 0.3, zeta-potential = −0.562 mV in pH 8 PB), and PLGA particles were 340 nm (PDI = 0.37).

### 2.5. Nanoparticles Encapsulation Efficiency and DNA Integrity

To test the integrity and amount of encapsulated DNA in PLGA nanoparticles, 0.2 mL nanoparticles dispersion in 10 mM Tris-HCl (pH 8) was extracted with 0.2 mL phenol: chloroform: isoamyl alcohol (25: 24: 1) and spun down at 12,100 g for 20 min. Then, 50 *μ*L of the aqueous layer was diluted with 250 *μ*L 10 mM Tris-HCl (pH 8) and extracted with 300 *μ*L CHCl_3_. The aqueous layer was separated by spinning down and analyzed by gel electrophoresis for DNA. To test the integrity of encapsulated DNA in the pH-responsive nanoparticles, nanoparticles (0.2 mL) in 10 mM Tris-HCl (pH 8) with heparin (1: 100 DNA to heparin), were extracted and analyzed as previously described with PLGA nanoparticles. To evaluate the encapsulation efficiency in the pH-responsive nanoparticles, the collected filtrate through the tangential flow (see previous section) was lyophilized and resuspended to determine the amount of DNA using 1% TAE agarose gel.

### 2.6. DNA Release From the Nanoparticles

 DNA-Cy5 nanoparticles were resuspended in phosphate buffer pH 7.4. The nanoparticles were left in a shaker at 60 rpm and 37°C. Aliquots were taken at different time intervals and spun down at 2,000 g and 4°C for 10 min. The supernatant was used to determine the fluorescence of released DNA-Cy5. After 24 hours, the particles were spun down and resuspended in phosphate buffer pH 5 to test the effect of pH on DNA release from the nanoparticles.

### 2.7. Transfection of DNA with Nanoparticles

 HCT116 cells were plated at ~50% density in a 24-well culture plate and allowed to attach overnight. Cells were then treated with nanoparticles encapsulating 50 to 100 ng of labeled or unlabeled DNA for 30 minutes, 1, 2, 3, or 4 hours in the presence of regular media with 10% serum. The media was then replaced with 500 *μ*L of fresh media in each well after washing to remove excess nanoparticles. For DNA-Cy5 analysis, the cells were immediately analyzed by fluorescence microscopy (Nikon and NIS Elements software) and flow cytometry (Accuri C6) by detecting fluorescence in the far red spectrum (670 nm). To analyze GFP expression, the cells were treated with nanoparticles for 4 hours,then the media was replaced and incubated for 48 hours. The cells were subsequently analyzed by fluorescence microscopy or flow cytometry (Accuri C6) to detect green fluorescence. For microscopy analysis, cells were placed in wells containing glass coverslips. For flow cytometry, cells were first trypsinized for 5 minutes followed by two washes with PBS and analyzed immediately. To test the requirement for low endosomal pH, cells were treated with Bafilomycin A1 at a final concentration of 300 nM prior to adding nanoparticles. The cells were then incubated for 4 hours, followed by replacement of media and incubation for 48 hours. 

## 3. Results and Discussion

### 3.1. DNA Encapsulation and Stability Study

Considering the obstacles to gene delivery, including DNA packaging, transport across the membrane, endosomal escape and transport into the nucleus, we aimed to demonstrate the effectiveness of our dual pH-responsive nanoparticles to meet these challenges. We first determined the stability and effectiveness of DNA encapsulation in the dual pH-responsive nanoparticles.

**Figure 2:**
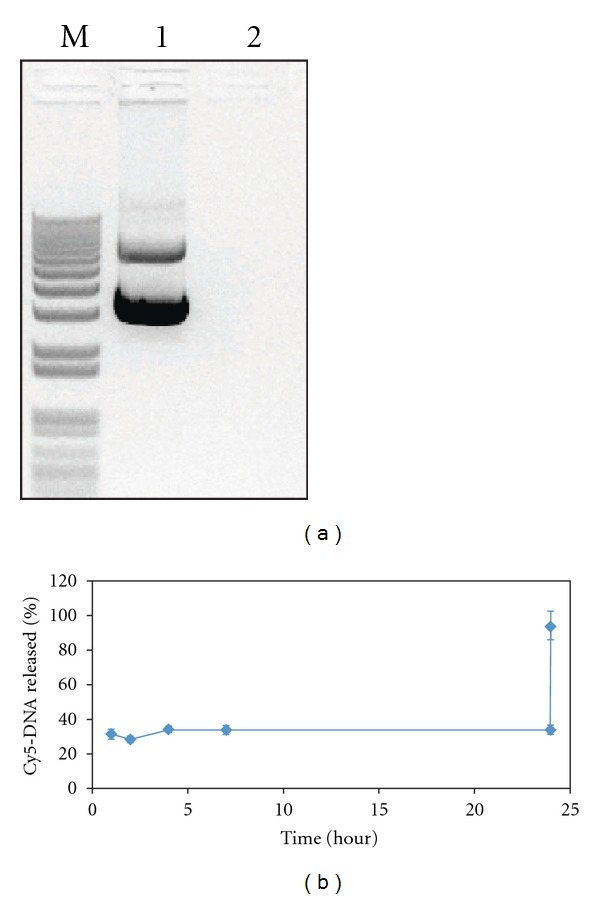
DNA encapsulation efficiency and release study. (a) DNA encapsulation efficiency was analyzed by comparing band intensity of control DNA (lane 1) to that of nonencapsulated DNA collected during the tangential flow filtration process (lane 2). (b) Cy5 fluorescence of released DNA from nanoparticles in buffer pH 7.4 over 24 hours followed by addition of pH 5 buffer. The pH changes to 5 at 24 hours.

The dual pH-responsive nanoparticles containing plasmid DNA were prepared with poly-*β*-aminoester ketal-2 using a double-emulsion method. The supernatant and washes of the preparations were kept and analyzed to estimate the percent of nonencapsulated DNA. The encapsulation efficiency was estimated to be approximately 100% since no DNA was detectable in these fractions (Figure 2(a)). The high encapsulation efficiency may be due to the high ratio of polymer to DNA in the nanoparticle-DNA formulation, approximately 133 : 1 polymer :  DNA wt. Also, this high encapsulation efficiency at pH 8 is not surprising because at this pH the polymer can carry enough positively charged groups to interact with the DNA efficiently (pKa = 6.7) [21]. This was not revealed on the measured zeta-potential (−0.562 mV) as the PVA residue (10–25%) is expected to shield the low positive charge at this pH [25]. To further demonstrate that DNA in the nanoparticle is well complexed, we mixed poly-*β*-aminoamide ketal, an analogous water-soluble polymer, with plasmid DNA using increasing polymer-to-DNA ratios and observed complete complexation at ratios beyond 2 Nitrogen : Phosphate ratio (corresponding to 1.4 : 1 polymer : DNA weight ratio) (Supplementary Figure 1 is available online at doi:10.1155/2012/291219).

Herein, the release of plasmid DNA from nanoparticles was monitored using Cy5-labeled DNA. The nanoparticles were very stable over a 24-hour period at the physiological pH of 7.4 (Figure 2(b)), which agrees with previous results on these nanoparticles [20]. There appears to be an initial release of DNA because of the change in pH from that of the preparation buffer (pH 8). Complete and immediate burst release of the nanoparticles occurred when the pH was dropped to 5, similar to the pH inside an endosome, as shown by the curve jump to 100%. The fast fragmentation of the polymer and release of DNA from nanoparticles occurs via a dual chemical response to low endosomal pH, which causes particles to undergo a hydrophobic-hydrophilic switch and leads to bulk and surface degradation. Particles were also treated with phenol/chloroform to extract the plasmid DNA, which was examined by gel electrophoresis to ensure that the encapsulation procedure did not affect the integrity of the plasmid. We observed very minimal degradation of plasmid DNA (Supplementary Figure 2).

### 3.2. Plasmid DNA Delivery and Expression of EGFP

**Figure 3:**
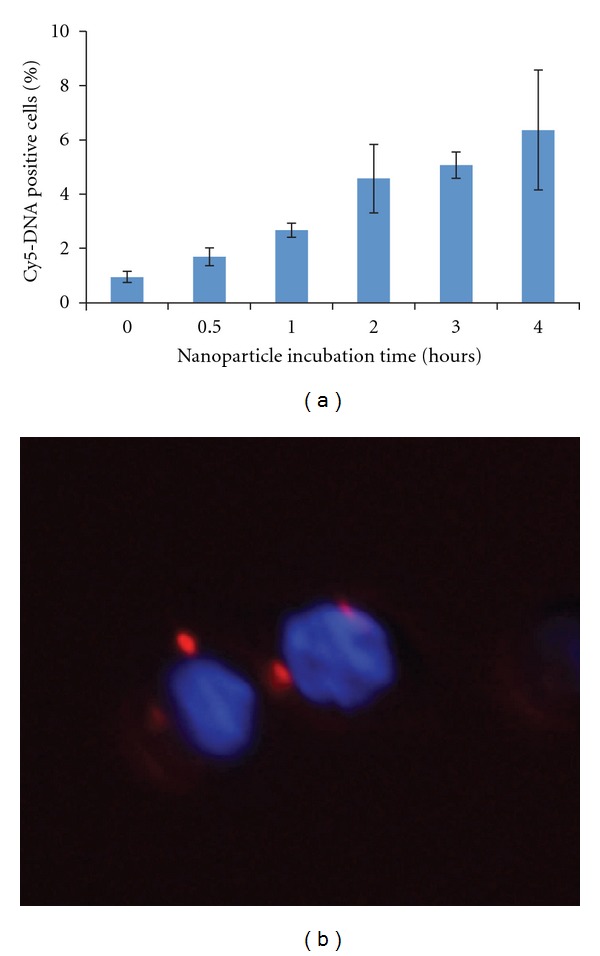
Cy5-labeled DNA delivery into cells via pH-responsive nanoparticles. HCT116 cells were incubated with pH-responsive nanoparticles containing Cy5-DNA for 0–4 hours and analyzed by flow cytometry (a) and microscopy (b) after 4 hours.

Next, we wanted to demonstrate that pH-responsive nanoparticles could cross the cell membrane and deliver DNA. Our previous toxicity studies on this polymer showed good cell tolerance up to 11 *μ*g/mL for 24 hours [21]; since we increased the concentration, we reduced exposure time to 4 hours. We were able to deliver up to 100 ng of pEGFP per well in 24-well plates without observing any changes in cell morphology or any other indication of cell death under the microscope. We analyzed cell uptake kinetics of nanoparticles using Cy5-labeled pEGFP DNA. The nanoparticles were allowed to be passively endocytosed by cells over 4 hours before flow cytometry analysis. Though only 5% of HCT116 cells showed detectable Cy5 fluorescence by 3 hours, which did not increase appreciably by 4 hours (Figure 3(a)), we believe most of the cells take up a small amount of particles that does not increase Cy5 signal appreciably. Microscopy analysis confirmed that Cy5-labeled pEGFP DNA was delivered inside cells and reached the periphery of the nucleus (Figure 3(b)). The uptake efficiency of the dual pH-responsive nanoparticles by HCT116 cells was very similar to PLGA nanoparticles (Supplementary Figure  3).

**Figure 4:**
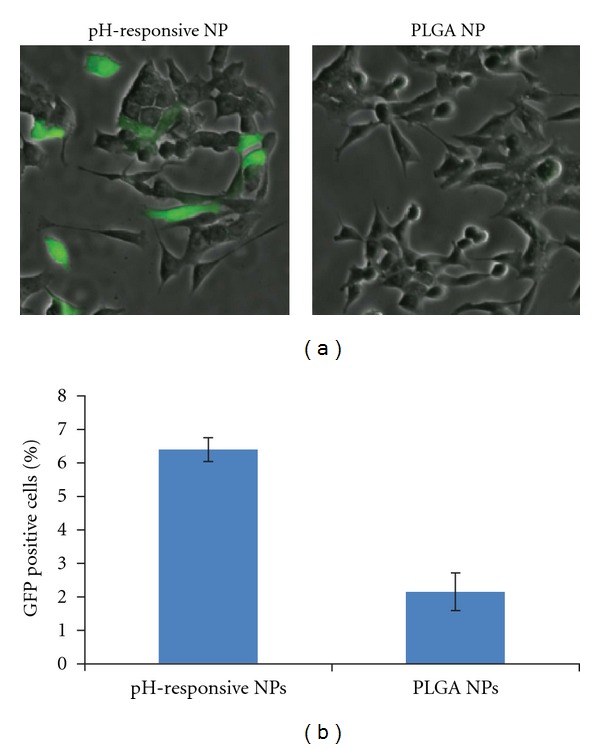
Expression of EGFP DNA with pH-responsive nanoparticles (NPs) compared to PLGA NPs: HCT116 cells were incubated with NPs for 3 hours, washed, and then incubated in media for 48 hours followed by (a) microscopy and (b) flow cytometry analysis.

Delivery of labeled plasmid into the cells via nanoparticles does not necessarily mean that EGFP will be expressed. To test expression and transfection efficiency, we incubated HCT116 cells with DNA-containing nanoparticles for 4 hours before washing to remove excess nanoparticles and incubating cells in fresh media for 48 hours. Cells treated with the dual pH-responsive nanoparticles produced intense green fluorescence when analyzed by microscopy. In contrast, cells treated with PLGA nanoparticles containing similar amounts of pEGFP DNA produced relatively low fluorescence (Figure 4(a)). Flow cytometry analysis revealed that approximately 6% of the cell population treated with the dual pH-responsive nanoparticles had fluorescence intensity higher than that of nontreated cells (Figure 4(b)), compared to 2% of cells treated with PLGA-DNA nanoparticles. The percentage of EGFP positive cells correlates well with the number of Cy5-positive cells, indicating that expression efficiency is high for cells that take up the nanoparticles. Additionally, comparing the intensity of EGFP-positive cells transfected with our nanoparticles or with an equal amount of DNA complexed with PEI, we show that our nanoparticles produce similar levels of EGFP expression within transfected cells (Supplementary Figure 4).

**Figure 5:**
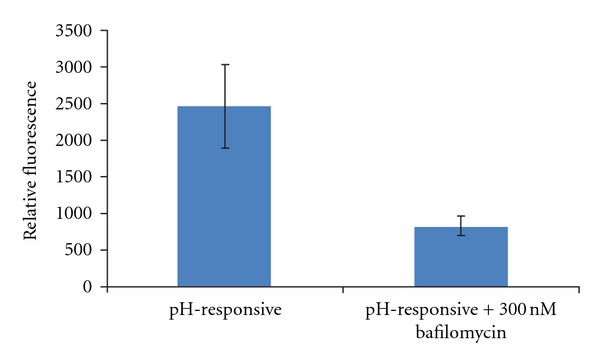
Dependence on endosomal low pH was analyzed by comparing transfection of nanoparticles in the presence or absence of 300 nM bafilomycin A1, a V-ATPase inhibitor.

Immediate burst degradation and release of DNA from the dual pH-responsive nanoparticles only occurs in an environment of low pH, similar to that present in endosomes. This low pH offers the appropriate stimulus to solubilize the polymer, resulting in an accelerated degradation via ketal hydrolysis. Bafilomycin A1, an inhibitor of V-ATPase, blocks the acidification of endosomes and has been previously used to characterize the mechanism of release of pH-dependent polyplexes and nanoparticles [26]. To verify that DNA release is pH-independent, the transfection efficiency of our dual pH-responsive nanoparticles was examined in the presence of 300 nM bafilomycin A1. EGFP expression via nanoparticle delivery in HCT116 cells, measured by mean flow cytometry fluorescence, was reduced by 66% in the presence of bafilomycin A1 relative to non-treated cells (Figure 5). This result indicates that expression of EGFP is dependent on the acidic endosomal pH in order for the nanoparticle to degrade rapidly and presumably cause an endosomal burst. The mechanism of action of the dual pH-responsive nanoparticles depends on the pH difference within endosomes and is thus an attractive system because particles can be maintained in stable conditions until they enter the targeted cells. Furthermore, the DNA integrity is maintained during nanoparticle degradation followed by endosomal escape. The exact mechanism for endosomal escape is still unclear, but we believe that the degraded nanoparticle causes significant instability in proton exchange and eventually bursts the endosome in a V-ATPase-dependent manner.

## 4. Conclusion

Our dual pH-responsive nanoparticles induce higher transfection efficiency than PLGA, a well-known slow-degradable polymeric material. This efficiency likely results from the nanoparticles' rapid surface and bulk degradation in response to endosomal pH as well as cells' tolerance for the polymer. The dual system forms a stable shield, as shown by Cy5 release at physiological pH, suggesting that it may be suitable for the protection of DNA from nuclease degradation. This stability, combined with its rapid fragmentation at low pH, means that DNA is released only if particles are endocytosed by cells. Our nanoparticles cause transfection, as demonstrated by Cy5 fluorescence following incubation of cells with particles containing labeled pDNA. The dual responsive nanoparticles produced a three-fold enhancement in EGFP expression over PLGA nanoparticles. Inhibition of V-ATPases using bafilomycin A1 demonstrates that expression of EGFP depends on low endosomal pH.

Our fast-release system offers multiple advantages over slow-release formulations. One significant example is that these nanoparticles may also be well suited for siRNA delivery. siRNA delivery via nanoparticles has already shown promising results using well-characterized polymers like PLGA [27]. Further experiments are underway to test if siRNA can be encapsulated and delivered. Furthermore, our advanced dual response nanoparticles offer new therapeutic possibilities, especially if combined with cell-type-specific peptides or antibodies to improved cellular entry and target specificity.

## Supplementary Material

Supplementary information includes complexation efficiency, DNA integrity, cellular uptake efficiency, and image analysis of transfected cells.
