# Supplementary material for: Triggered Rapid Degradation of Nanoparticles for Gene Delivery
Source: J Drug Deliv. 2012 Jun 19;2012:291219. doi: 10.1155/2012/291219 (PMC3388356; doi:10.1155/2012/291219)
Supplement: Supplementary file 1 — Supplementary information includes complexation efficiency, DNA integrity, cellular uptake efficiency, and image analysis of transfected cells. [file 291219.f1.pdf]

Supplementary Data:

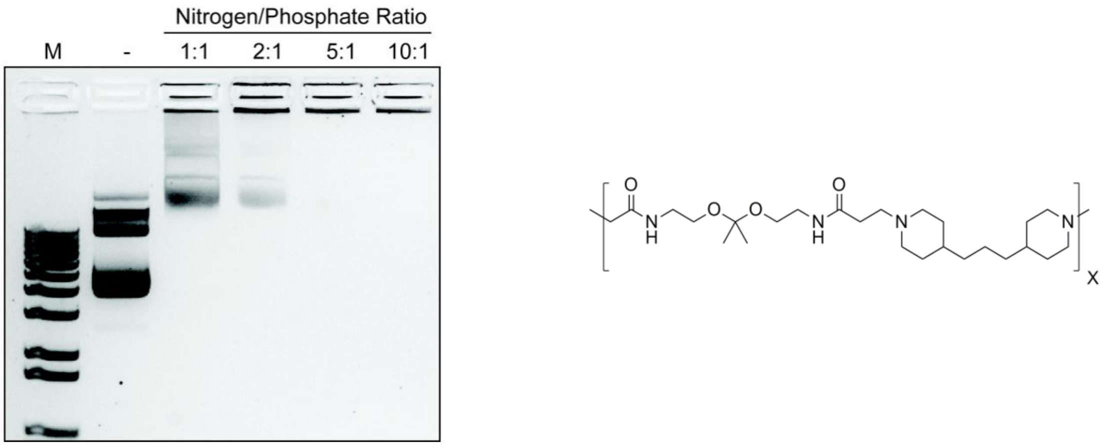

Supplementary Figure 1: Complexation efficiency of polyß-aminoamide ketal (structure presented) **with DNA** (to predict that for pH-responsive nanoparticles). Gel electrophoresis of DNA combined with varying quantities of polyß-aminoamide ketal. N/P ratios were manipulated by varying the volume of 3 nM nitrogen/µL polyß-aminoamide ketal (0.07% W/V in 10 mM Tris (pH 7.5)) mixed with 8 µL of 0.5 µg/µL DNA, diluting with 10 mM Tris pH 7.5 to adjust the DNA concentration to 50 ng/µL before loading them into the gel.

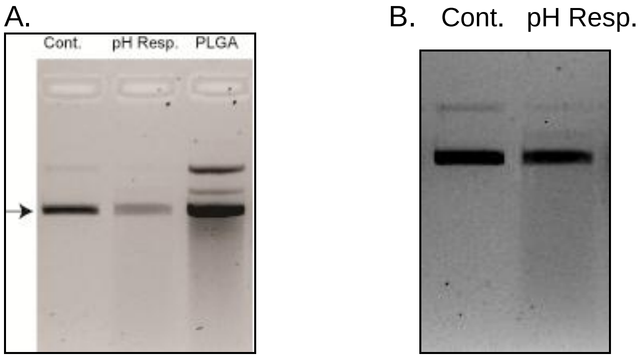

Supplementary Figure 2: Integrity of DNA extracted from PLGA and pH responsive particles by gel electrophoresis. Lane1: Control DNA, Lane 2: DNA extracted from pH responsive particles using heparin and phenol/chloroform followed by chloroform, Lane 3: DNA extracted from PLGA nanoparticles using phenol/choloroform followed by chloroform. Arrow indicates supercoiled plasmid. (A) Lanes were not normalized to the control and are shown simply to illustrate that plasmid DNA after extraction remains largely intact. (B) Zoom in version of lanes 1 and 2 from A with higher intensity. The encapsulation efficiency of DNA in PLGA nanoparticlers was 32.43% ± 5.31.

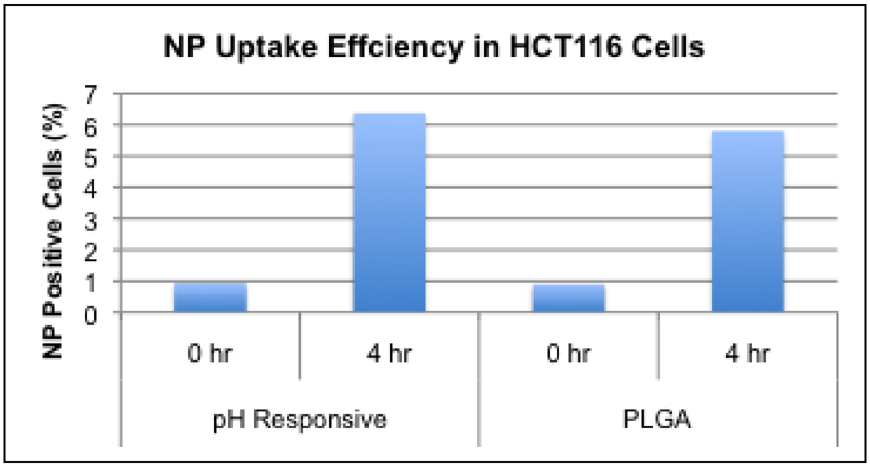

Supplementary Figure 3: Uptake efficiency in HCT116 cells. Cells were incubated with either pH sensitive or PLGA nanoparticles encapsulating a fluorescent reporter and analyzied by flow cytometry. The pH sensitive and PLGA nanoparticles have similar uptake efficiencies of about 6.27% and 5.8% respectively.

A.

B.

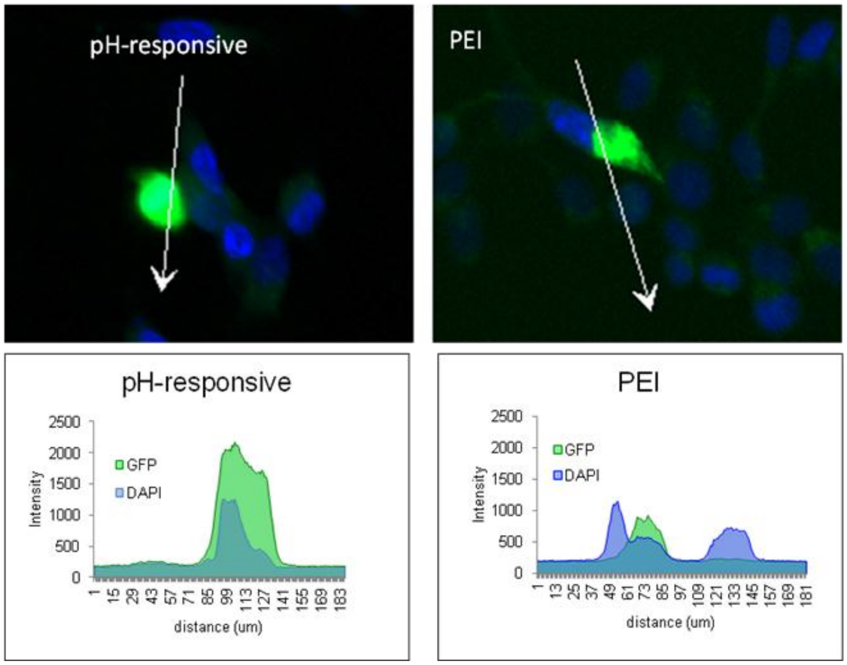

Supplementary Figure 4: Intensity comparison after transfection with the same total amount of DNA (100ng) using pH-responsive nanoparticles or PEI in 24 well plates of HCT116 cells. (A) Microscopy analysis and (B) quantification of the fluorescence intensity through the line shown in the microscopy images.
